# Simulation of Normally-Off Vertical GaN MOSFET with a Novel Enhanced Sidewall Channel by Selective Area Growth

**DOI:** 10.3390/mi16010105

**Published:** 2025-01-17

**Authors:** Jiyao Du, Taofei Pu, Xiaobo Li, Liuan Li, Jinping Ao, Hongwei Gao

**Affiliations:** 1 School of Automation and Electrical Engineering, Shenyang Ligong University, Shenyang 110159, China; 2 Science and Technology Development Corporation, Shenyang Ligong University, Shenyang 110003, China; 3 School of Mechanical Engineering and Automation, Northeastern University, Shenyang 110819, China; 4 Lancomm Semiconductor (Hangzhou) Co., Ltd., Hangzhou 310018, China; fbc_ptf@126.com (T.P.); lixiaobo166@163.com (X.L.); 5 State Key Laboratory of Superhard Material, Jilin University, Changchun 130012, China; 6 Engineering Research Center of Internet of Things Technology Applications, Jiangnan University, Wuxi 214122, China; jpao1800@jiangnan.edu.cn

**Keywords:** GaN MOSFET, vertical device, sidewall channel, selective area growth, simulation

## Abstract

In the present study, a novel normally-off vertical GaN MOSFET with an enhanced AlGaN/GaN channel on the sidewall has been proposed using the technology computer-aided design (TCAD) simulation. By using the selective area growth process, the trench structure and the enhanced sidewall channel are formed simultaneously, which is beneficial to enhance the conduction capability compared with the conventional trenched MOSFET. It demonstrates that a proper hole concentration and thickness of the p-GaN layer are key parameters to balance the threshold voltage, on-state resistance, and off-state breakdown voltage, resulting in the highest Baliga’s figure of merit value. Furthermore, a p-GaN shield layer is also adopted as a junction termination extension to modulate the electric field around the trench bottom. By optimizing the device parameters, a normally-off GaN MOSFET with good performance is designed.

## 1. Introduction

GaN power devices are mainly categorized into lateral and vertical conduction routes [1,2]. The lateral diodes and transistors based on AlGaN/GaN heterojunctions have demonstrated excellent electrical performance and have been gradually introduced as commercial products. Also, many types of research focusing on monolithic integration with complementary logic and gate drivers are also reported to minimize the switching loss and gate protection [3,4]. However, the device size increases rapidly with increased power level, leading to inefficient material utilization and current extraction difficulties [5]. In addition, electrons in a two-dimensional electron gas (2DEG) channel are captured by surface traps under a high off-state electric field, resulting in a current collapse effect that degrades device performance and long-term reliability [6]. The exploration of vertical GaN power devices is expected to overcome the challenges faced by lateral devices, which is important to promote the development of the GaN industry. Furthermore, the normally-off (or enhance-mode) field-effect transistors (FETs) that can completely pinch off the 2DEG channel under a gate bias (*V_g_*) of 0 V are more preferable for power devices due to the simple gate drive circuit design and inherent fail-safe property.

At present, three main technology routes have been developed to obtain vertical GaN field-effect transistors (FETs) in terms of device structures, namely, the current aperture structure [7], fin metal–oxide–semiconductor FET (MOSFET) structure [8], and trenched MOSFET structure [9,10]. Current aperture transistors utilize a localized p-GaN as a current-blocking layer and a regrown AlGaN/GaN heterostructure as a channel. The presence of heterostructure allows for high channel mobility but makes it difficult to achieve normally-off operation [11]. This GaN device with current aperture structures fabricated by multiple-energy implantation shows an output current of 190 A/cm^2^ under a gate voltage of 1 V, while the threshold voltage (*V_th_*) was −6 V (normally on operation) [7]. The current of the fin MOSFET passes through a submicron fin-shaped vertical channel and is controlled by the surrounding gate metal to avoid the difficulties of p-GaN growth. At a *V_g_* of 0 V, the channel is depleted by the work function difference between the gate metal and GaN, resulting in the normally-off performance. On the other hand, the depletion width decreases, and a conduction channel forms when *V_g_* is increased to higher than *V_th_*. The weakness is that it needs to downscale the size of the fin to approximately 450 nm for a *V_th_* of +1V and an output current of 15 kA/cm^2^, resulting in electric field crowding and heat dissipation challenges. Trenched MOSFETs based on the NPN GaN epitaxy layer structure form the inversion channel between the gate dielectric and p-GaN, which is beneficial to remove the internal parasitic junction transistor and improve the integrated density. This route presents many advantages, such as high *V_th_* (>3 V), high breakdown voltage, and process simplicity. However, unlike the lateral transistor on AlGaN/GaN, the channel of vertical trenched MOSFET exists at the p-GaN/dielectric interface on the sidewall of the trench, and the intrinsic low mobility of p-GaN (approximately 25 cm^2^/Vs) and surface damage caused by etching result in a high on-state resistance (*R_on_*).

Many different technologies have been developed to enhance the channel mobility of trenched MOSFETs. Treatment with KOH or TMAH solution can repair the lattice damage at the etched surface but can only enhance the device channel mobility to a limited range [12,13]. A two-step process, including simple acid cleaning and (NH_4_)_2_S passivation, is also proposed to fabricate trench MOSFETs with a high-quality interface. The vertical GaN MOSFET presents a threshold voltage of 3.15 V and *R_on_* of 1.93 mΩ·cm^2^ [14]. Other reports demonstrate that regrowth of a thin GaN layer (approximately 10 nm) on the sidewalls of the trench can separate the p-GaN and dielectric, resulting in an improved channel mobility (with the *R_on_* decreased to 2.6 mΩ·cm^2^), a *V_th_* of 3 V, and a withstand voltage of 1 KV. However, the interface charge caused by etching damage resulted in a lower *V_th_* than the theoretical value [15]. Panasonic proposed a regrown p-GaN/AlGaN/GaN structure on the sidewalls of the trench to significantly improve the channel mobility (approximately 1690 cm^2^/Vs). A *V_th_* of 2.5 V and a *R_on_* of 1.0 mΩ·cm^2^ were realized by increasing the inclination angle of the sidewall to reduce the polarization charge in the AlGaN/GaN interface [16]. Therefore, separating the channel from p-GaN is an effective route to enhance channel mobility, but the lattice damage caused by etching hinders the improvement of channel mobility and threshold voltage [17]. We previously realized a laterally trenched gate in GaN MOSFET without etching damage by using the selective area growth (SAG) method [18,19]. It is worth noting that a thin layer of low Al-content AlGaN/GaN heterojunction exists on the sidewall during the regrowth process, which is beneficial to enhance channel mobility. However, the related work on vertical-trenched MOSFET using the SAG method has not been reported.

In this work, a normally-off vertical GaN MOSFET has been proposed by using the TCAD P-2019.03 simulation, in which the AlGaN/GaN channel on the sidewall is enhanced. A SAG method is used for forming the trench structure and the enhanced sidewall channel, which is beneficial to enhance the conduction capability compared with the conventional trenched MOSFET. By the simulation investigation, the appropriate hole concentration and thickness of the p-GaN layer are key parameters to make a trade-off between the *V_th_*, *R_on_*, and off-state breakdown voltage (*V_br_*), which results in the highest Baliga’s figure of merit value. Moreover, as junction termination extension, the p-GaN shield layer is also adopted to regulate the electric field around the trench bottom. By optimizing the device parameters, normally-off GaN MOSFET with great performance is designed.

## 2. Design Models and Calibration

**Figure 1:**
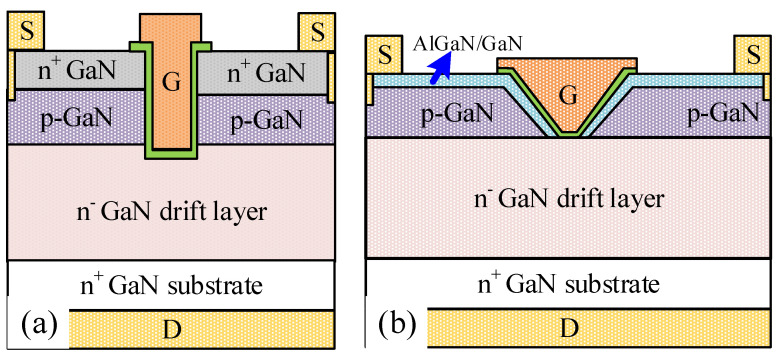
Schematic structures of the conventional trenched MOSFET (**a**) and trenched MOSFET with an enhanced sidewall channel structure (**b**).

Figure 1 shows the schematic structures designed by quasi-stationary TCAD, including the conventional trenched MOSFET (CT-MOS, Figure 1a) and the proposed trenched MOSFET with an enhanced sidewall channel (ESC-MOS, Figure 1b). The vertical GaN devices are designed on a free-standing GaN wafer with a thickness of 350 μm and an electron concentration of 1 × 10^18^ cm^−3^. The GaN drift layer is n-type doped with a thickness of 15 μm and an electron concentration of 7 × 10^15^ cm^−3^. For the CT-MOS, a 300 nm p-type GaN layer (hole concentration of 2 × 10^18^ cm^−3^ and mobility of 25 cm^2^/Vs) and a 200 nm n-type GaN layer (electron concentration of 2 × 10^18^ cm^−3^ and mobility of 500 cm^2^/Vs) are stacked on the drift layer. The trench depth is approximately 500 nm. For the ESC-MOS, a p-type GaN layer (thickness of 100–500 nm, hole concentration of 1 × 10^17^ cm^−3^–6 × 10^18^ cm^−3^, and mobility of 25 cm^2^/Vs) and an Al_0.25_Ga_0.75_N/GaN (25/25 nm) heterojunction are stacked on the drift layer. The 2DEG in the heterojunction channel possesses an electron concentration of 6.51 × 10^12^ cm^−2^ and mobility of 2000 cm^2^/Vs. On the other hand, the electron concentration and mobility are set to be 3.25 × 10^12^ cm^−2^ because of the semi-polar plane and 2000 cm^2^/Vs on the sidewall, respectively. The gate dielectric is Si_3_N_4,_ with a thickness of 10 nm. The source, drain, and gate electrodes are set to ohmic contact with the semiconductor materials local beneath. The dimensions of the device are *L_g_/L_gs_/L_gd_* = 2.45/0.02/15.01 μm. In addition, common piezoelectric polarization, doping dependence mobility, high field saturation mobility, Auger recombination, Shockley–Read–Hall (SRH) recombination, and impact ionization (avalanche) models were added to the simulation process. The output current–voltage (*I_d_*–*V_d_*), transfer characteristics (*I_d_*–*V_g_*), and off-state current–voltage (*I_d_*–*V_d_*) curves, as well as the electric field distributions, were obtained.

## 3. Results and Discussion

**Figure 2:**
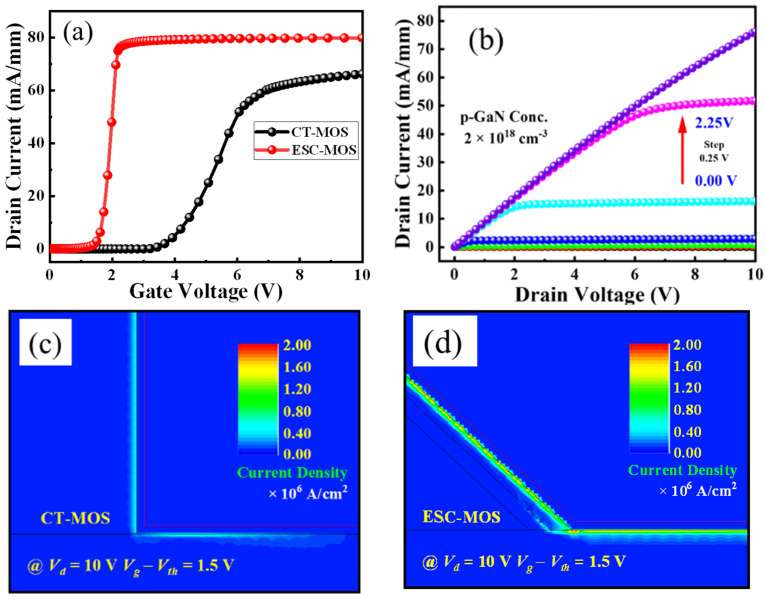
Transfer curves (**a**) of the CT-MOS and ESC-MOS devices. (**b**) is the typical output characteristic of ESC-MOS with 300 nm p-GaN (hole concentration of 2 × 10^18^ cm^−3^). (**c**,**d**) are the corresponding forward current distributions with an overdrive voltage of 1.5 V.

Firstly, we investigated the effect of the enhanced sidewall channel on the electrical characteristics compared with the conventional CT-MOS (Figure 2). The p-GaN thickness and hole concentration are set to 300 nm and 2 × 10^18^ cm^−3^, respectively. Observed from the transfer curves as shown in Figure 2a, both devices are normally-off operations with a positive *V_th_*. The sidewall 2DEG channel of the ESC-MOS device (300 nm p-GaN with a hole concentration of 2 × 10^18^ cm^−3^) provides more electrons, which weakens the depleted capability of p-GaN partly, resulting in the *V_th_* negative shifting. On the other hand, the current density increases from approximately 60 to 80 mA/mm. The corresponding output curves of ESC-MOS present good pinch-off characteristics with the gate voltage varying from 0 to 2.2 V (a step of 0.25 V) (Figure 2b). The current distributions of both devices are simulated under the same overdrive voltage (*V_g_- V_th_*) and drain voltage (Figure 2c,d). Obviously, the sidewall 2DEG channel is beneficial to enhance the carrier concentration and current density.

**Figure 3:**
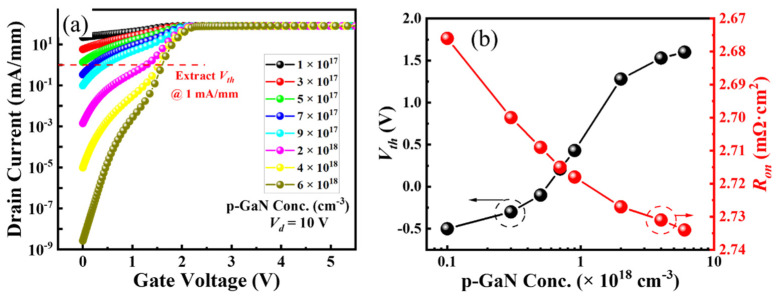
Transfer curves in semi-log scale (**a**) and deduced threshold voltages and on-state resistances (**b**) of the ESC-MOS devices with different hole concentrations of the p-GaN layer.

**Figure 4:**
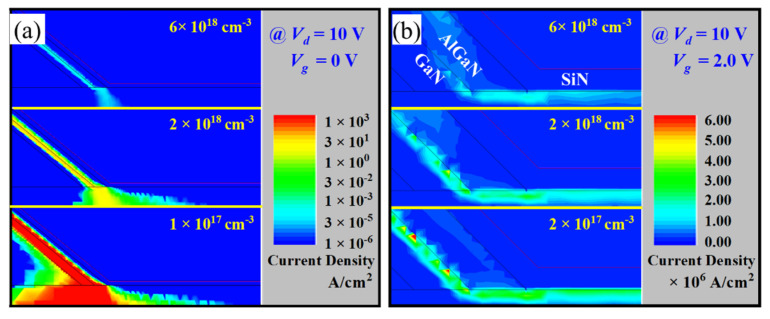
Forward current distributions at a gate voltage of 0 V (**a**) and 2 V (**b**) of the ESC-MOS devices with different hole concentrations of the p-GaN layer.

Figure 3a shows the effects of hole concentration in the p-GaN on the transfer curves of the ESC-MOS devices. The *V_th_* is deduced at a current density of 1 mA/mm and summarized in Figure 3b, showing a positive shift from −0.5 V to +1.6 V. In addition, the drain leakage current in the subthreshold region is relatively higher for the p-GaN with a small hole concentration and then can be suppressed obviously for the higher hole concentration. The variation in *V_th_* and drain leakage current can be explained by the current density distributions at a gate voltage of 0 V, as shown in Figure 4a. For the hole concentration of 1 × 10^17^ cm^−3^, the device is normally-on operation with a large leakage current along the sidewall because the p-GaN does not deplete the sidewall channel entirely. The depletion effect is enhanced when the hole concentration is higher than 5 × 10^17^ cm^−3^, obtaining the normally-off operation. However, the output current density is also decreasing with the increasing hole concentration and increasing the on-state resistance, as shown in Figure 3b simultaneously. Observed from the current density distributions at a gate voltage of 2 V as shown in Figure 4b, the total current density in the case of fully turn-on is the sum of sidewall current and the MOS channel current at the trench bottom.

**Figure 5:**
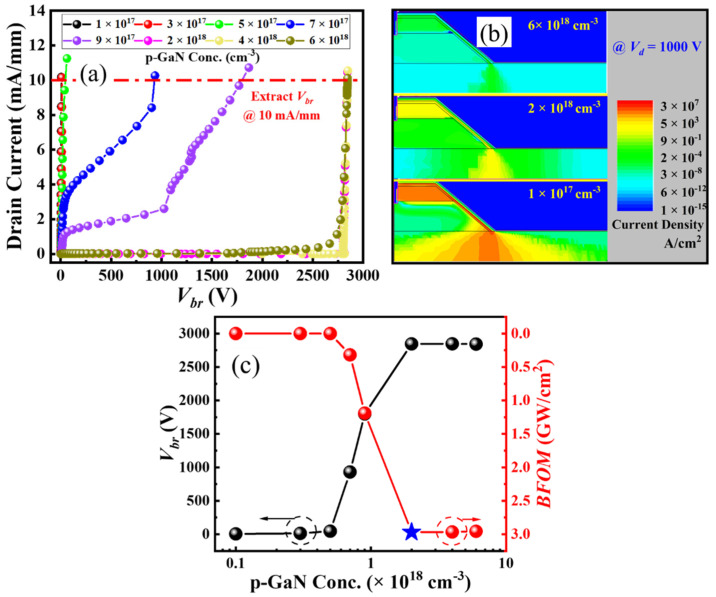
Off-state drain current (**a**), electric field distributions (**b**), and deduced breakdown voltages (**c**) of the ESC-MOS devices with different hole concentrations in the p-GaN layer.

The off-state drain current is simulated versus drain voltage at a gate voltage of 0 V (Figure 5a). The *V_br_* of the ESC-MOS devices are defined at the criteria of current density reaching 10 mA/mm. The *V_br_* increases drastically with the increasing hole concentration first and then saturates at a medium hole concentration of 2 × 10^18^ cm^−3^ (Figure 5c). The off-state drain current density distributions of the typical devices at the drain voltage of 1000 V are simulated, as shown in Figure 5b. When the hole concentration is relatively small, the depletion effect of the p-GaN is not enough to pinch off the sidewall channel and causes the large leakage current. When the hole concentration increases gradually to 2 × 10^18^ cm^−3^, the depletion regions of p-GaN extend into the sidewall channel and increase the *V_br_*. We also confirm that the electric field in the dielectric around the trench bottom is approximately 7 MV/cm, which is smaller than the critical electric field of the dielectric. It demonstrates that the device with a medium hole concentration of 2 × 10^18^ cm^−3^ presents the highest Baliga’s figure of merit (BFOM) (BFOM = *V_br_*^2^/*R_on_*) (Figure 5c). Therefore, we choose the hole concentration of 2 × 10^18^ cm^−3^ as the optimal value for the following simulations.

**Figure 6:**
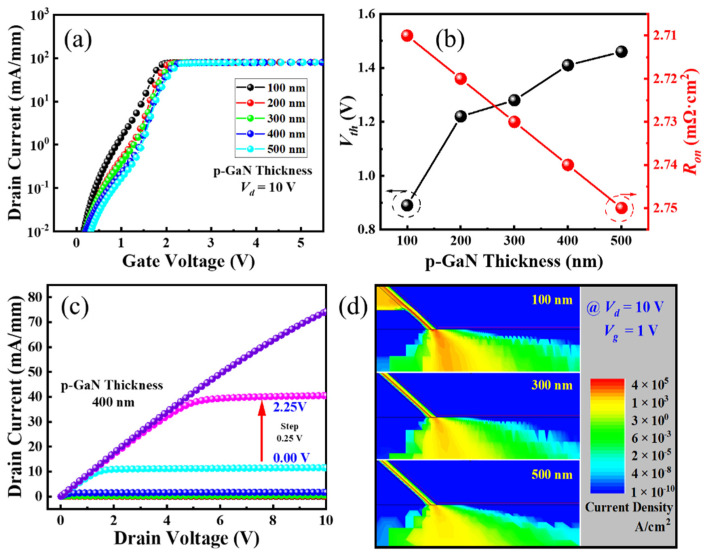
Electrical performance of ESC-MOS devices with different p-GaN layer thicknesses. (**a**) Transfer curves in semi-log scale and (**b**) deduced threshold voltages and on-state resistances. (**c**) the typical output characteristic with 400 nm p-GaN (hole concentration of 2 × 10^18^ cm^−3^). (**d**) the forward current distributions under a *V_g_* of 1 V for three p-GaN thicknesses.

The thickness of the p-GaN is further evaluated based on the electric properties of the ESC-MOS devices (Figure 6). The *V_th_* is deduced on the transfer curves at a current density of 1 mA/mm (Figure 6a), showing a slight increase from 0.9 to +1.5 V (Figure 6b). The possible reason for the variation in *V_th_* can be attributed to the increase in gate length because the channel is formed on the p-GaN/GaN/AlGaN interface. Then, a thicker p-GaN depletes more sidewall channels and decreases the current density (Figure 6d), which can also be confirmed from the slight increase in *R_on_*, as shown in Figure 6b.

**Figure 7:**
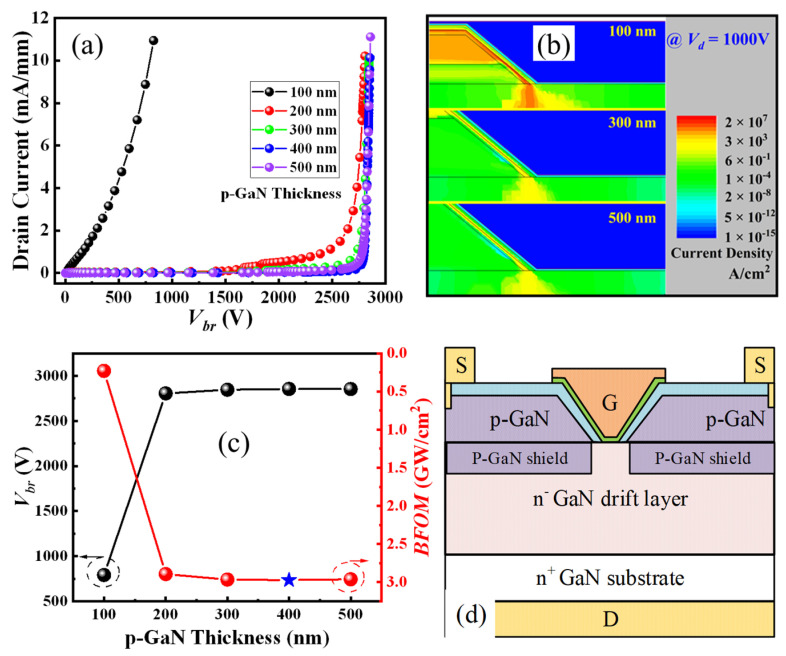
Off-state drain current (**a**), electric field distribution (**b**), and deduced breakdown voltages (**c**) of the ESC-MOS devices with different p-GaN layer thicknesses. And the schematic structures of ESC-MOSFET with a p-GaN shield layer (**d**).

The off-state drain current is relatively high for the device with 100 nm p-GaN, while it is suppressed drastically with a thicker p-GaN (Figure 7a). Therefore, the *V_br_* of the ESC-MOS devices increases slightly firstly when the thickness increases from 100 nm to 200 nm and then saturates at approximately 2750 V. Observed from the electric field distributions under *V_br_*, as shown in Figure 7b, the 100 nm p-GaN is depleted totally while it is only partially depleted for the thickness larger than 300 nm. Therefore, the deduced BFOM values present an increasing trend versus the p-GaN thickness (Figure 7c). Taking into account the *V_th_* and *V_br_*, we think that 400 nm is the optimum parameter, and the corresponding output characteristic confirms a good pinch-off and maximum drain current of approximately 80 mA/mm (Figure 6c).

**Figure 8:**
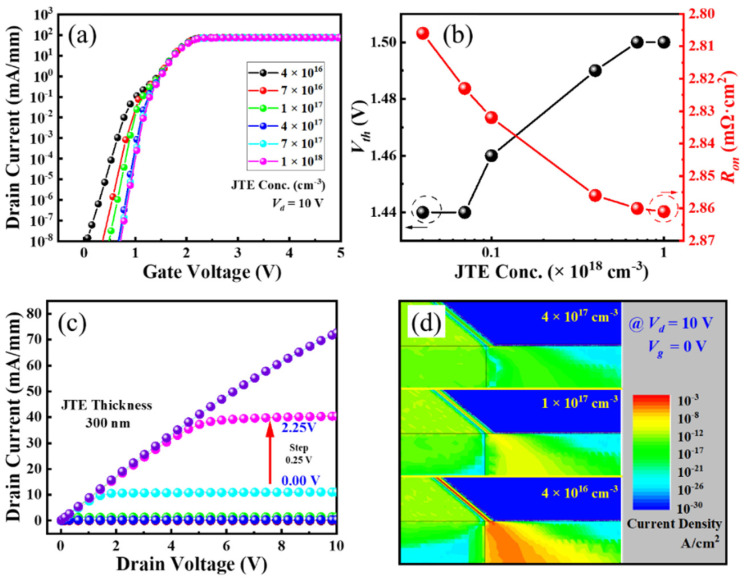
Transfer curves in semi-log scale (**a**), deduced threshold voltages and on-state resistances (**b**), optimized output characteristics (**c**), and forward current distributions (**d**) of the ESC-MOS devices with different hole concentrations of the p-GaN shield layer.

As discussed above, the off-state drain current usually crowds around the corner of the trench bottom. Therefore, we adopt a p-GaN shield layer local beneath the p-GaN as the junction termination extension to suppress the leakage current path, as shown in Figure 7d. The hole concentration in the p-GaN shield layer is also a key parameter for the ESC-MOS devices (Figure 8a). As shown in Figure 8b, the *V_th_* deduced from the transfer curves shows a slight positive shift from 1.44 to 1.50 V when the hole concentration varies from 4 × 10^16^ cm^−3^ to 1 × 10^18^ cm^−3^. The current density distributions demonstrate that the depletion regions formed by the p-GaN shield layer expand into the drift layer by the increasing hole concentration, which suppresses the current leakage of the subthreshold region but also increases the *R_on_* (Figure 8d). Then, under the same drain voltage, it is necessary to increase the gate voltage to obtain the same drain current density.

**Figure 9:**
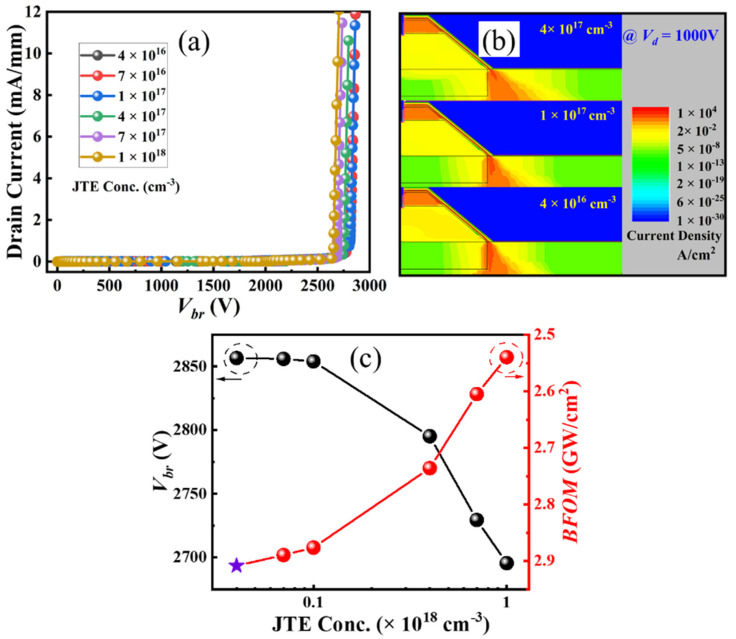
Off-state drain current (**a**), electric field distribution (**b**), and deduced breakdown voltages (**c**) of the ESC-MOS devices with different p-GaN shield layer hole concentrations.

The off-state drain current also presents a mild hole concentration dependency, with the *V_br_* showing a slight decreasing trend versus hole concentration (Figure 9a). The effect of this p-GaN shield layer is comparable with that of the junction termination extension used in the power devices. The increasing hole concentration eliminates the depletion region in the p-GaN and extends it into the drift layer below the trench (Figure 9b). It is difficult for the p-GaN to control this region, resulting in a slightly increasing leakage current. It demonstrates that the device with a relatively lower hole concentration of the p-GaN shield layer presents the highest BFOM value, as shown in Figure 9c. The corresponding output characteristic of the hole concentration of 4 × 10^16^ cm^−3^ confirms a good pinch-off and maximum drain current of approximately 70 mA/mm when the gate voltages are swept from 0 to 2.25 V (with a step of 0.25 V) (Figure 8c).

**Figure 10:**
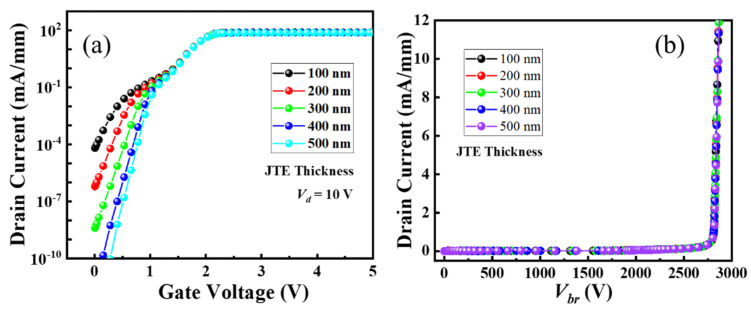
Transfer curves in semi-log scale (**a**) and off-state drain current (**b**) of the ESC-MOS devices with different p-GaN shield layer thicknesses.

Figure 10 shows the transfer curves of the ESC-MOS devices with different thicknesses of the p-GaN shield layer. The *V_th_* are deduced on the transfer curves and are comparable for all the devices (Figure 10a), but the current leakage of the subthreshold region is suppressed obviously when the thickness is more than 300 nm. Furthermore, the off-state *V_br_* of the devices is also comparable for all the devices (Figure 10b). Therefore, we think that the thickness of 300 nm is the optimum parameter for the p-GaN shield layer.

**Figure 11:**
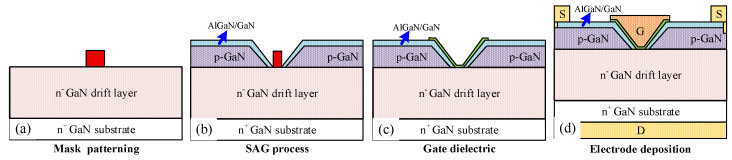
Main fabrication process steps of the ESC-MOSFET. (**a**) deposition and patterning of the mask, (**b**) SAG of the p-GaN and AlGaN/GaN heterojunction, (**c**) deposition of gate dielectric layer, and (**d**) deposition of electrode metal.

Finally, the main fabrication processes are shown in Figure 11. After the epitaxy of the GaN drift layer on the freestanding GaN substrate, the mask (SiO_2_, for example) is deposited and patterned. Then, the GaN wafer is sent back to the chamber after clear cleaning for the selective area growth of p-GaN and AlGaN/GaN heterojunction. The discrepancy between the polar and non-polar plane, the trenched-gate structure with a beveled sidewall, is formed during the SAG. Therefore, the AlGaN/GaN heterojunction can also cover the sidewall to enhance the channel mobility. The growth of p-GaN with high doping concentration is a slight challenge for the GaN device. However, great progress has been achieved in recent years, which is beneficial to realizing the SAG of p-GaN with good electric properties in the future. The SAG p-GaN gate of the AlGaN/GaN high electron mobility transistor has a dimension of 10 μm. Compared with Schottky gate HFETs, the SAG p-GaN gate HFETs show more positive *V_th_* and better gate control ability. The SAG method paves a promising way for achieving p-GaN gate normally-off AlGaN/GaN HFETs without dry etching damage [20]. Similarly, the selective area growth of p-GaN is also used to fabricate a junction barrier Schottky diode with the dimension of p-GaN being about 1 μm. Good rectification characteristics are also confirmed [21]. It is worth noting that the dimension of the mask region is relatively smaller, which makes the growth of p-GaN with good electric performance much easier. After the growth of the gate dielectric, the source, drain, and gate electrodes are deposited by using the conventional lift-off method.

## 4. Conclusions

In summary, a normally-off vertical GaN MOSFET with an enhanced sidewall channel has been proposed using the TCAD simulation. Compared with the conventional trenched MOSFET, the sidewall-enhanced channel provides extra electrons to enhance the conduction capability at the cost of *V_th_* negative shifting. For the p-GaN layer, an increasing hole concentration and thickness present a stronger depletion effect on the sidewall channel, resulting in a higher *V_th_*, higher *R_on_*, as well as higher *V_br_*. The optimal hole concentration and thickness for the p-GaN layer are determined to be 2 × 10^18^ cm^−3^ and 400 nm, respectively. Furthermore, it demonstrates that a p-GaN JTE structure with higher hole concentrations is beneficial to suppressing the leakage current in the subthreshold region while decreasing the *V_br_*. On the other hand, a relatively greater thickness can suppress the leakage current in the subthreshold region without decreasing the *V_br_*. The optimal hole concentration and thickness of the p-GaN JTE are determined to be 4 × 10^16^ cm^−3^ and 300 nm, respectively. Those results provide some valuable references for the design and fabrication of normally-off GaN MOSFETs with good performance.

## Data Availability

The original contributions presented in this study are included in the article. Further inquiries can be directed to the corresponding author.
